# The emotional labor of end-of-life care work: Findings of a mixed methods study in Hong Kong

**DOI:** 10.1017/S1478951526102417

**Published:** 2026-04-28

**Authors:** Biyun Li, Margo Turnbull, Carol Yu, Xiaoyan Wu

**Affiliations:** 1 Department of English and Communication, The Hong Kong Polytechnic University, Hung Hom, Hong Kong, China; 2 Ip Ying To Lee Yu Yee School of Humanities and Languages, Saint Francis University, Tseung Kwan O, Hong Kong, China

**Keywords:** Emotional labor, EOL care practitioner, end-of-life, multidisciplinary workforce, Hong Kong

## Abstract

**Objectives:**

This study explored how end-of-life (EOL) care practitioners in Hong Kong engaged in emotional labor while fulfilling their professional roles in a Chinese cultural context.

**Methods:**

A sequential explanatory mixed-methods design was employed. A quantitative survey (*n* = 32) using validated scales that measured emotional job demands and emotional labor strategies was followed by in-depth interviews (*n* = 11) with EOL care practitioners from diverse disciplines. Survey data were analyzed using descriptive statistics, while interview transcripts underwent thematic analysis.

**Results:**

EOL care practitioners reported high emotional job demands, with deep acting being their preferred emotional labor strategy over surface acting. Three key themes emerged related to: (1) balancing emotional involvement and professional boundaries; (2) employing strategic emotional engagement; and (3) navigating cultural beliefs and family dynamics. This multidisciplinary workforce developed sophisticated practices to manage their emotions authentically while establishing protective psychological boundaries. These practices integrated the provision of emotional support with the navigation of tensions between Chinese cultural values and professional responsibilities.

**Significance of results:**

This study used mixed-methods to explore how traditional values were integrated into the everyday care practices of EOL practitioners in Hong Kong. The findings contribute to an innovative and culturally sensitive framework for exploring emotional labor in EOL care contexts. This is useful in both Chinese and multicultural care contexts.

## Introduction

### Background

Demand for community-based end-of-life (EOL) care has grown in recent decades as a result of the interaction of multiple factors, including population aging and increases in chronic diseases (Gengler 2023; Turnbull et al. 2023). EOL care policy has evolved to increasingly focus on the improvement of the quality of life for individuals facing anticipated death due to life-limiting conditions or significant functional deterioration (Turnbull et al. 2023). Services are seeking to provide holistic physical, psychological, spiritual, and medical care (Brighton et al. 2019). This care involves a diverse multidisciplinary workforce, with physicians, nurses, support workers, spiritual counselors, pharmacists, and others playing equally important roles (World Health Organization 2020) in the provision of comprehensive support to care recipients and their families throughout the stages of dying and death (Brighton et al. 2019; Thompson et al. 2022). In this study, we use the term “EOL care practitioners” to encompass this professional and workforce diversity.

The very nature of EOL care work means it involves a significant portion of relational and interpersonal communication. EOL care practitioners are actively engaged in interaction “work” as they provide company, empathy, and support to clients and families (Turnbull et al. 2022). The complexity of EOL care practitioners’ roles often requires them to mediate client and family dynamics, regulate their own emotional states, and respond empathetically to others’ feelings as well as maintain professional standards (Thompson et al. 2022). This ongoing and varied engagement with the emotions associated with loss and grief constitutes a type of emotional “work” that can be conceptualized as a form of emotional labor in EOL care (Funk et al. 2017; Umubyeyi et al. 2024). Emotional labor refers to the processes through which individuals work to manage and express feelings and emotions as they seek to fulfill the requirements of a job (Hochschild 1983). This type of work arises in situations where employees are required by their employing entity to regulate their emotions to foster desired emotional states in others, such as comfort, trust, or satisfaction among clients or customers (Dutta et al. 2020).

Effective emotional labor can enable individual workers to meet job requirements, particularly in roles involving high levels of engagement with clients (e.g., health care workers and teachers). However, the sustained effort of emotion regulation can impact workers’ well-being, increasing their vulnerability to emotional exhaustion and burnout (Aiello and Tesi 2017). How individuals manage their emotional labor is particularly relevant in EOL care, as practitioners must anticipate and navigate complex and changing relationships and communicative environments. The concept of emotional labor provides a useful perspective for the examination of how practitioners balance managing their own emotions while meeting the needs of clients and families (Hochschild 1983; Funk et al. 2017).

Despite international developments in EOL care policy and care provision, limited research has explored the emotional labor of the multidisciplinary group of EOL care workforce, particularly in East Asian settings (Dutta et al. 2020). Cultural contexts profoundly impact societal approaches to understanding, communicating about, and handling death, and thus, research across diverse locations is needed (Dutta et al. 2020; Turnbull et al. 2023). This article contributes to these areas of work by presenting a study that explored how EOL care practitioners in the Chinese cultural context of Hong Kong engage in emotional labor while fulfilling their professional roles within multidisciplinary teams. Our findings provide insights for workforce and policy development across the many contexts in which practitioners and/or clients may approach EOL from different linguistic, ethnic, and cultural perspectives (Dutta et al. 2020).

### EOL care policy and practice in Hong Kong’s cultural context

Hong Kong’s approach to EOL care is shaped by traditional Chinese cultural perspectives on death and dying. These beliefs help to shape more indirect approaches to communication about death and emphasize family-centered decision-making (Turnbull et al. 2023). This cultural context distinguishes Hong Kong’s EOL care development from Western models and makes it an important case study for exploring how Asian societies integrate contemporary health care approaches with traditional cultural values (Yee and Law 2022).

Since 2000, the Hong Kong government has introduced several key EOL care initiatives, including the Advance Directives (AD) and “dying in place” policy framework (Food and Health Bureau 2019). This was accompanied by a public consultation in 2019, which explored public awareness, the feasibility of various EOL care options, and potential policy and legislative changes needed to support EOL care (Yee and Law 2022). The primary settings for EOL care delivery in Hong Kong are Residential Care Homes for the Elderly (RCHEs), operated by non-governmental organizations, non-profit organizations, and private operators (Social Welfare Department 2024). Unlike hospital environments, RCHEs are designed as living spaces focused on quality of life, where staff develop profound emotional and social relationships with residents and their families (Yee and Law 2022). These EOL care settings provide unique insights into the interplay between emotional labor demands, cultural beliefs about death, and policy-led service changes.

## Methods

### Research design and sample

This research employed a sequential explanatory mixed-methods design (Creswell and Creswell 2018) consisting of 2 phases: a quantitative survey followed by a series of qualitative semi-structured interviews. The quantitative phase provided descriptive insights into EOL practitioners’ emotional job demand and emotional labor strategies, while the qualitative phase offered an in-depth exploration of their lived experiences. Purposive sampling was used to recruit EOL care practitioners in Hong Kong via the existing social networks of the second and third authors (M.T. and C.Y.). To be eligible, participants needed to be currently employed in jobs in EOL care services in Hong Kong. All research activities were conducted in Cantonese. This research was approved by the authors’ Institutional Review Board (HSEARS20210126005-04). Written information about the research was provided to participants, and electronic informed consent was obtained from all participants prior to participation. Participants were reminded that they could withdraw from the research at any stage.

### Instruments

An online survey encompassing 2 validated scales was used in phase 1 of the research. Both scales were originally validated in English and had been previously adapted for use in Hong Kong (Wong and Law 2002; Yin 2015; Yang et al. 2019). For this study, scales underwent a forward–backward translation process into Chinese to ensure cultural and linguistic equivalence. All items in the survey were scored on a Likert scale (1 = strongly disagree; 5 = strongly agree).

#### Emotional Job Demands scale

This 4-item scale measured the emotional demands participants experienced in their professional roles in the EOL care setting. It was adapted from the validated scales developed by Wong and Law (2002) and Yin (2015). Higher scores reflect higher perceived emotional demands. An example item is, “I have to use my emotions and behaviors to create a reassuring climate for my clients and their families.” The Cronbach’s alpha in the reported study was 0.86, indicating good internal consistency and reliability of the scale for measuring emotional demands in this context.

#### Emotional Labor Strategy scale

This 16-item scale was adapted for use in the context of EOL care work from the Teacher Emotional Labor Strategy scale developed by Yang et al. (2019). This scale measured 4 subdimension strategies related to emotional labor: (1) surface acting – faking emotions or hiding felt emotions to display expected work emotions (e.g., “I fake the emotions I show when dealing with clients or their families.”); (2) deep acting – modifying one’s true feelings to display expected work emotions (e.g., “I try to actually experience the emotions that I must show to clients or their families.”); (3) expression of naturally felt emotions – displaying emotions that arise spontaneously without modifications (e.g., “The emotions I show clients or their families match what I spontaneously feel.”); and (4) emotion termination – actively suppressing emotional displays (e.g., “Where there is a disagreement with the clients or their families, I will serve according to their requirements without any emotional change.”). A higher score on a subscale indicates more frequent use of that specific strategy. The Cronbach’s alpha in this study was 0.91, demonstrating excellent internal consistency and reliability of the adapted scale for measuring emotional labor strategies in EOL care contexts.

### Data collection and analysis

Survey data were collected in November 2022. An online survey was distributed to EOL care practitioners, and 32 out of the 37 individuals who clicked on the survey link completed it (completion rate: 86%). Table 1 provides an overview of the survey respondents’ demographic details. The survey data were analyzed using descriptive statistics, which served as a preliminary step to provide contextual information related to perceptions of emotional labor.

**Table 1.** Characteristics of survey participants (*n* = 32)

Characteristic	Number	Percentage (%)
Age in years		
18−25	5	16
26−40	15	47
41−59	9	28
>60	3	9
Gender		
Female	26	81
Male	6	19
Education level		
University	24	75
High school	3	9
Primary school	1	3
Others	4	13
Employment pattern		
Full-time	29	91
Part-time	2	6
Temporary/casual	1	3
Years of experience		
<5	13	41
5−10	7	22
11−15	2	6
>15	10	31

Eleven EOL care practitioners from the survey participant pool volunteered to participate in phase 2 of the research. The participants consisted of both early-career (<5 years) and highly experienced (>20 years) practitioners across different roles (registered nurses, social workers, administrators, and clinic staff). Table 2 summarizes the characteristics of interview participants.

**Table 2.** Characteristics of interview participants (*n* = 11)

	Gender	Age group	Years of experience	Role
P1	Male	35−44	>10 years	Clinical staff, social worker
P2	Male	45−54	>20 years	Social worker manager
P3	Female	35−44	4 years	Registered nurse
P4	Female	35−44	>20 years	Registered nurse
P5	Female	35−44	20 years	Registered nurse
P6	Female	35−44	10 years	Registered nurse
P7	Female	25−34	>5 years	Registered nurse
P8	Female	25−34	5 years	Registered nurse
P9	Male	55−64	>30 years	Staff at Hospital Authority
P10	Male	>65	>40 years	Operation manager
P11	Female	35−44	23 years	Administrator

Following the interview guide (Appendix A), semi-structured interviews were conducted in Cantonese by the third author (C.Y.) via phone calls. The interview guide was developed to explore EOL care practitioners’ lived experiences in relation to emotional labor. The guide comprised 5 open-ended questions organized to establish participants’ professional background and motivations, assess the interpersonal demands of their role, explore training and support in emotional communication, and elicit in-depth narratives about emotional experiences and coping strategies. The interviews were audio-recorded with consent and lasted between 10 and 41 minutes, with an average duration of 28 minutes. The interview recordings were transcribed verbatim in Chinese and were verified for accuracy by the interviewer. The Chinese transcripts were analyzed using thematic analysis (Clarke and Braun 2017). The first author (B.L.) read and re-read transcripts to identify initial codes and conducted inductive coding, which was reviewed by the fourth author (X.W.). Codes were grouped into broader categories and refined into themes through discussion within the broader team. Discrepancies in coding were resolved through discussion within the research team. Illustrative quotes have been translated into English for inclusion in this article. Backward–forward translation was done by the first and fourth authors.

Triangulation was conducted during analysis by integrating quantitative and qualitative findings (Ivankova et al. 2006; Othman et al. 2020) in alignment with our research objectives. The quantitative results showed the distribution and prevalence of emotional labor phenomena and informed the interpretation of qualitative findings. The qualitative data analysis primarily focused on exploring the in-depth and nuanced aspects of participants’ emotional experiences.

## Results

This section reports the integrated findings from the quantitative and qualitative data. Survey results are summarized in Appendix B.

### Balancing emotional involvement and professional imperatives

Overall, results of the quantitative survey confirmed that participants engaged in significant emotional labor within the course of their daily work. Most participants reported that their role required them to spend a large amount of time with others (e.g., clients and families) and that they must manage their emotions and behaviors to create a reassuring climate for clients and families. Around 90% reported that they have to think from the perspective of their clients to perform their job well. These findings underscore the high emotional job demands placed on EOL care practitioners, as their role requires consistent emotional engagement, interpersonal sensitivity, and the ability to manage complex relational dynamics.

Qualitative analysis added depth to these findings by illustrating the challenges practitioners faced in balancing emotional involvement with professional imperatives. Participants often described the need to engage deeply with clients and families while maintaining professional boundaries. They needed to deal with difficult conversations about death and dying in a way that was viewed not only as respectful and empathetic, but also as fulfilling the professional imperatives and objectives of their jobs. For example, one participant explained how she carefully tailored her communication to match what she perceived as a family’s readiness to receive sensitive information:

If we notice that the family or the client is not very clear about it (EOL care) or does not accept it, we will explain it slowly and see how much they can understand. We will sometimes not be able to go all the way at once…we will take some time to develop a relationship with them…then we will give more information to them every time we see them. (P3, Female, Registered Nurse)

Some participants acknowledged the emotional toll of their work and accepted emotional involvement as an inevitable or expected part of their role, but they also emphasized the importance of boundaries. One participant shared the following insights:

I probably expect myself to feel sad when my job is end-of-life related. So, I allow myself to be unhappy and sad, but I wouldn’t say it’s a burden or I feel stressed. (P2, Male, Social Worker Manager)

This sentiment reflects the effort made by the individual to process emotions without becoming overwhelmed. Another participant noted that emotional demands can become overwhelming and linked this with the potential for burnout:

Burnout happens. Sometimes when we are deeply involved and we are feeling the emotions of clients and their families, it affects us and can make us feel low. I had worked in this field for such a long time, and at a point, I felt burnt out and left the field for a few years. (P4, Female, Registered Nurse)

Other participants highlighted that while emotional engagement is a necessary component of their work, they needed to protect their own well-being to sustain their ability to care for clients. For example, one participant shared:

I like to go hiking and go back to nature, which I think is great. Nature makes me feel like there are so many amazing things in this world and I am so tiny. Then I would forget about my worries. (P2, Male, Social Worker Manager)

This quote draws attention to how the individual engaged in activities outside work to actively keep perspective and recharge emotionally. Meanwhile, peer support and supervision also played a critical role in helping practitioners cope with challenges and stresses. One participant explained:

When some cases are emotionally heavy, we may talk about them with colleagues…We got peer support and sometimes supervision to guide us. For example, we share with others when we face difficulties and ask them what to do…I think supervision is important. (P1, Male, Social Worker)

This statement shows that supervision and peer support created a collaborative and supportive environment. Peer support specifically provided important opportunities to share their emotional load, validate their feelings, and gain practical advice from colleagues who understood their challenges. Structured supervision was also important as it provided a framework for reflection and guidance.

### Strategic emotional engagement in EOL care

The survey results show that deep acting was the most frequently used emotional labor strategy. Over 80% of the respondents reported making deliberate attempts to feel the emotions they needed to display, and only 22% reported using surface acting. Around half of the participants reported employing the emotion termination strategy (that is, actively suppressing emotional displays) and modifying their practices at times to suit requirements from clients and families without emotional change in the face of disagreement. This suggests that individuals may view maintaining emotional stability during disagreements as professionally appropriate. These survey findings demonstrate that respondents valued emotional authenticity in their interactions with clients and families.

The preference for emotional authenticity was also reflected in the interviews. The practitioners described the deep emotional connections they developed with clients and the sadness they felt when those clients passed away. A participant shared:

Like some clients, I already took care of them for a very long time and we built up a deep relationship…we were like family. The moment they died, I felt very sad and sometimes it is hard to detach myself from those sad feelings. (P6, Female, Registered Nurse)

This quote shows how practitioners’ emotional labor extended beyond their interactions with clients and families and included the personal sorrow they experienced from losing clients they had cared for over time. Interviewees also described nuanced emotional labor strategies that integrated authentic emotional experiences with effective psychological boundaries. This approach allowed practitioners to maintain professional effectiveness while acknowledging genuine emotions. One participant commented:

Yes, (emotional stress is part of my job) but I can detach myself from it. Even if I feel sad, as I noted previously, the sadness won’t bother me for days and nights or cause insomnia. (P2, Male, Social Worker Manager)

Rather than merely suppressing or modifying emotions, this practitioner demonstrates the ability to experience authentic emotions while establishing temporal and psychological boundaries.

### Navigating cultural beliefs and family dynamics

Interview participants’ responses highlighted the cultural challenges they negotiated to provide EOL care in Hong Kong, where death remains a sensitive and taboo topic due to the traditional Chinese philosophies that view discussing death as inauspicious. Many families avoid conversations about death and dying due to deeply ingrained beliefs that discussing it might bring bad luck. This taboo creates barriers for practitioners. The participants remarked how cultural norms could inhibit open discussions about death and dying, making it difficult for practitioners to address sensitive issues directly with clients and their families:

Sometimes we can’t get to the point to deliver messages about death. They are Chinese after all. They may not accept death as it’s a taboo topic. (P3, Female, Registered Nurse)

In our Chinese society, it is still taboo to talk about death. However, as practitioners, we believe that departing from the world is an inevitable stage of life. (P7, Female, Registered Nurse)

In the second statement, the participant’s use of “our Chinese society” signifies a shared cultural perspective of death between herself and clients or families. The need to balance these perspectives with professional beliefs or objectives may add extra complexity to the practitioners’ emotional work.

The traditional Chinese value of filial piety can complicate these conversations, as families often take on the responsibility of making decisions rather than deferring to individual clients for decision-making. This dynamic can cause conflict within families, particularly when family members have different views on the best course of action. One participant shared:

If there are several children, not all of them think the same way. Some of them insist that everything should be done – that the mother must be saved no matter what. Others say that if intubation is needed, then just go ahead with it as they don’t want their mother to suffer, but for others, they thought that intubating her would mean she would end up starving to death, as if they themselves were causing the harm. (P5, Female, Registered Nurse)

This quote provides an example of how familial decision-making in EOL care may be influenced by traditional Chinese beliefs about the afterlife, as exemplified by another participant’s reference to the cultural concept – “Even if we die, we should die a full ghost rather than a hungry ghost” (P3, Female, Registered Nurse). Interview participants indicated that they needed to balance honoring cultural values with advocating for the client’s comfort and well-being.

## Discussion

Our findings present a novel exploration of the emotional labor of EOL care practitioners in Hong Kong. Our analysis identifies both the nature of this work as well as the sophisticated strategies they developed to navigate complex emotional demands. This complexity is evident in practitioners’ accounts of developing deep and authentic connections with clients and families over time, which makes client deaths particularly impactful on their emotional well-being. This aligns with previous research identifying the ongoing engagement with loss and grief as a main form of emotional labor in EOL care (Funk et al. 2017; Wu et al. 2022; Umubyeyi et al. 2024). However, our findings extend beyond documenting these demands by exploring how practitioners strategically manage them. They actively calibrated emotional engagement and developed protective boundaries while maintaining relational authenticity as well as sustaining professional performance during repeated exposure to death and dying. Participants also identified particularly helpful institutional and personal support mechanisms, including peer support, organizational supervision, and self-care. Our study also expands on existing research by highlighting how practitioners manage emotional labor within Hong Kong’s specific cultural and institutional context. Participants described how they often had to navigate traditional Chinese cultural taboos around death while fulfilling their professional responsibilities. They described particular challenges managing family dynamics shaped by filial piety, where adult children often make health care decisions for elderly parents and may resist discussions about terminal conditions. Critically, rather than experience these tensions passively, practitioners proactively worked within cultural frameworks using indirect communication approaches and establishing rapport and trust with families. This extends earlier observations by Turnbull et al. (2023) and Yee and Law (2022) regarding the distinctive challenges of implementing EOL care effectively in different cultural contexts. The emotional management and regulation strategies practitioners employed may also apply to other Asian contexts facing similar tensions in terms of aging populations, declines in family-based care models, and a concomitant growth in institutional care. While institutionally organized care enables comprehensive services, it embeds practitioners within cost-efficacy imperatives and resource constraints that shape emotional labor.

The findings of this research have implications for policy, practice, and education in EOL care. Policymakers are encouraged to recognize emotional labor as central to a worker’s capacity to provide quality care and allocate supporting resources. Tensions and interconnections between emotional labor, cultural norms, and professional practice also influence the everyday practices of workers, and support at the institutional level is imperative. Health care organizations can establish comprehensive supporting mechanisms, while training programs can develop self-protection strategies and culturally informed communication frameworks supporting practitioners’ navigation of professional and cultural demands.

Our study has two key limitations. First, the sample sizes of the quantitative and qualitative phases were relatively small due to pandemic constraints during the data collection period and the sensitive nature of EOL care in Hong Kong. Second, our cross-sectional design captures practitioners’ experiences at a single point in time, which restricts the examination of how emotional labor might evolve over the course of a career in EOL care. Future work can address these limitations by recruiting a larger sample of participants and utilizing a longitudinal research design.

## Conclusion

This study provides valuable insights into the emotional labor of multidisciplinary EOL care practitioners in Hong Kong’s Chinese context. The practitioners employed emotional management strategies that balanced authenticity with self-protection, which was identified as a means of preventing potential burnout. The professional practice of this multidisciplinary workforce was characterized by the integration of emotional care as fundamental to their work, while they simultaneously navigated tensions between Chinese cultural values and professional responsibilities to deliver what they perceived as high-quality EOL care in publicly funded settings. This research marks a first step towards addressing the gap identified in the broader field of research regarding the limited understanding of EOL care and practitioners’ experiences of emotional labor in East-Asian contexts.

## Appendix A

### Interview guide

1. How long have you been working in this industry? Can you tell me about your role at work?
2. Why do you work in the EOL field? What do your family/friends think about your work?
3. Does your job involve a lot of interpersonal contact with clients/families?
4. Have you attended training courses specifically about communication with clients/families?
5. Do you find your work highly emotional? How do you cope with these job-related emotions both inside and outside work? How would you describe your experience of emotional labor at work?

## Appendix B

**Table:** Emotional Job Demand Scale (*n* = 32)

		5 Strongly Agree	4	3	2	1 Strongly Disagree
1	To perform my job well, I have to spend most of my time interacting with others (e.g., clients, families, and colleagues).	11	17	4	0	0
2	To perform my job well, I have to be considerate and think from the point of view of my clients and colleagues.	16	13	3	0	0
3	To perform my job well, I have to spend a lot of time on every client whom I instruct.	8	15	9	0	0
4	I have to use my emotions and behaviors to create a reassuring climate for my clients and their families.	11	12	9	0	0

**Table:** Emotional Labor Strategy Scale (*n* = 32)

		5 Strongly Agree	4	3	2	1 Strongly Disagree
**Surface acting**						
1	I fake the emotions I show when dealing with clients or their families.	1	6	5	12	8
2	I just pretend to have the emotions I need to display for my job.	1	6	10	10	5
3	I put on a “mask” in order to display the emotions I need for the job.	3	9	10	9	1
4	I put on an act in order to deal with clients or their families in an appropriate way.	3	10	7	9	3
**Deep acting**						
1	I try to actually experience the emotions that I must show to clients or their families.	6	22	3	0	1
2	I make an effort to actually feel the emotions that I need to display towards clients or their families.	7	20	4	0	1
3	I work hard to feel the emotions that I need to show to clients or their families.	6	20	3	1	2
4	I work on developing the feelings inside of me that I need to show to clients or their families.	4	23	2	2	1
**Expression of naturally felt emotions**						
1	The emotions I show clients or their families match what I spontaneously feel.	4	18	7	2	1
2	The emotions I show clients or their families come naturally.	4	17	10	0	1
3	The emotions I express to clients or their families are genuine.	4	17	9	1	1
**Emotion termination**						
1	Where there is a disagreement with the clients or their families, I will serve according to their requirements without any emotional change.	2	14	12	3	1
2	When clients or their families disapprove of my service, I will choose silence.	0	5	16	9	2
3	I have no feelings when clients’ or their families’ demands are too much or difficult to satisfy.	0	4	8	13	7
4	When clients or their families ask for too much, I just work by the organizers’ rules without emotions	0	3	15	12	2
5	When I cannot communicate with clients or their families normally, I will choose not to communicate, and without any emotions.	0	3	11	12	6
